# Visualization-supported dialogues in the Baltic Sea Region

**DOI:** 10.1007/s13280-019-01250-6

**Published:** 2019-09-21

**Authors:** Tina-Simone Neset, Julie Wilk, Carlo Navarra, René Capell, Alena Bartosova

**Affiliations:** 1 Department of Thematic Studies – Environmental Change, Linköping University, 58183 Linköping, Sweden; 2 Swedish Meteorological and Hydrological Institute (SMHI), 60176 Norrköping, Sweden

**Keywords:** BONUS MIRACLE, Geographic visualization, Good governance, Participatory processes, Stakeholder dialogues

## Abstract

This study explores visualization-supported dialogues with water management and ecosystem stakeholders from four catchments in Sweden, Latvia, Germany and Poland. An interactive visualization tool was designed to present information regarding modelled effects of chosen future pathways including different measures that address ecosystem issues under present and future scenarios of land use and climate change, and estimated benefits and costs of the measures. This paper assesses if and how visualization-supported dialogues hinder or support key components of good governance of water and ecosystem management among expert stakeholders. We discuss challenges and opportunities related to the tool and dialogue design, and performance of dialogues. Results from a cross-case workshop indicate that the form and functionality of the tool contributes to participation, empowerment, accessibility and flexibility, while dialogue design is instrumental for encouraging trust and inclusion of local knowledge and competence.

## Introduction

The Baltic Sea, surrounded by nine nation states, is an ecosystem exposed to multiple stressors, such as warming and nutrient pollution (Reusche et al. 2018). Collaborative efforts are essential to address such complex environmental issues and contribute to good governance. Effective approaches to governing nutrient-related challenges require that trade-offs are jointly addressed by environmental experts responsible for flood control, protection of vulnerable areas, biodiversity conservation, human health and biosecurity. As the use of ecosystem services intensifies in the Baltic Sea Region (BSR), the role of individuals and groups becomes increasingly important and management decisions become increasingly contested, as found in other heavily used European ecosystems (Turner et al. 2016). Although good governance is a goal to which many processes and products aspire, it is generally poorly defined and has been criticized for this ambiguity (McCall and Dunn 2012). Governance determines how decisions are undertaken and implemented, and good governance refers to a robust, transparent and fair relationship between governing bodies and those that are governed (McCall and Dunn 2012).

Stakeholder dialogues and participation in decision-making have been promoted in water management since the recommendation of Integrated Water Resource Management in the Dublin principles of 1992 (Snellen and Schrevel 2004). It is believed to enhance river basin management by encouraging discussion and consensus building for better quality decisions, engaging and supporting human and social capital to implement decisions and increase their legitimacy (Carr 2015). Participation is often seen as a characteristic of group processes strongly linked with ownership of information and processes that increase the likelihood of implementing joint decisions (McCall and Dunn 2012). Policy-making that strives to achieve equity and governance requires participation that is genuine, inclusive and effective (Cagnin et al. 2011). In participatory processes, where issues are addressed over longer periods of time, individuals holding different perspectives and objectives can gain trust and respect for those of other stakeholders and increase their collaborative learning. Stakeholder dialogues have been found effective in supporting collective actions (Maiello et al. 2013; Yuen et al. 2013; Chu et al. 2016), allowing stakeholders to explore multiple framings of an issue and when the issues being discussed are as unpredictable as those related to environmental change (Tyler and Moench 2012).

This study relates to governance that encourages the implementation of effective measures to reduce nutrient enrichment and flood risks in the BSR. As comprehensive assessments of the sustainability of socio-ecological systems and how they relate to ground conditions require a multi-sectoral stakeholder approach (Angelstam et al. 2013), expert stakeholders were consulted to gain a rich picture of the specific contexts of nutrient- and flood-related issues that demonstrate key points of concern and contention. These issues became the point of departure from which to guide the design of visualization-supported dialogues in which experts could use an interactive tool to explore, inform and share different perspectives and priorities. While several studies have assessed the role of landscape, geographic and information visualization, as well as participatory GIS, to effectively communicate scientific content and context and increase stakeholder engagement (e.g. Salter et al. 2009; Sheppard et al. 2011; Sheppard 2012; Rød et al. 2015; Neset et al. 2016), this study takes a wider perspective on visualization-supported dialogues, assessing the interactive tool but also dialogue design and usage of the tool in stakeholder dialogues. This paper presents an analysis of the design of the tool and dialogues with expert stakeholders from four catchments of the BSR: Berze (Latvia), Reda (Poland), Helge (Sweden) and Selke (Germany). We aim to assess if and how such visualization-supported dialogues hinder or support key components of good governance of water and ecosystem management among expert stakeholders in these four case areas.

## Analytical framework

Visualization-supported dialogues refer to the integration of participatory processes and interactive visualization methodology (e.g. Sheppard 2012; Meier et al. 2014; Neset et al. 2019). Visual representations have the ability to enable an immediate understanding of complex and heterogeneous data (Tufte 1997), and numerous studies have pointed towards the potential to increased engagement of stakeholders in participatory events that feature visual representations and interactive visualization tools (e.g. Nicholson-Cole 2005; Shaw et al. 2009; Sheppard 2012, 2015; Rød et al. 2015; Bohman et al. 2015). A high degree of interactivity in a tool, enabling users to explore the information provided, has the potential to support a reflexive process that is required for strategic planning and decision-making (Beers et al. 2010; Vervoort et al. 2010). Geographic visualization techniques can facilitate an interactive exploration to increase transparency and to avoid misinterpretations as well as over-simplifications (MacEachren and Kraak 2001, Rød et al. 2015). Geospatial representations that visualize sustainability status have the potential to assist stakeholders in defining and verifying information and setting targets (Axelsson et al. 2013). Previous studies have outlined characteristics that need to be met to ensure good governance, ethical correctness and strong knowledge systems. Sheppard (2012) outlined a code of ethics with seven criteria for designing, using and evaluation of landscape visualization in participatory settings. *Accuracy* refers to how well a visual representation simulates the actual appearance of an object e.g. landscape. *Representativeness* refers to how well typical and important perspectives and conditions, e.g. the case areas or scenarios are represented, *visual clarity* and *presentation* refer to the degree one communicates content in a clear and neutral way. *Accessibility* and *interest* relate to the degree users can access material and how well it holds their interest. *Legitimacy* relates to both transparent and defensible presentation and the accuracy of the visualization.

Similar criteria have been identified in studies of participatory processes, planning tools and collaborative learning. Cash et al. (2003) highlighted the importance of creating knowledge systems where the process and outcomes are credible (contain sufficient scientific adequacy), salient (are relevant to decision makers’ needs) and legitimate (are unbiased, fair and respectful of stakeholders’ diverse values and beliefs). Sheng (2009) listed eight major characteristics of good governance: participation, consensus-oriented, accountability, transparency, responsiveness, effectiveness and efficiency, equity and inclusiveness, and following the rule of law. McCall and Dunn (2012) identified a number of components to which participatory spatial planning tools and processes could contribute across five fundamental principles of good governance: legitimacy, respect, equity, competence and accountability.

We selected relevant criteria used in studies of GIS participatory processes to evaluate how the use of expert stakeholder dialogues in this study contribute to McCall and Dunn’s (2012) principles of good governance. Legitimacy includes *participation* (participation in all phases of the process from issue exploration and assessment to process and tool design), *empowerment* (the ability for the process and tool to provide confidence and meet stakeholder needs), *ownership* of product and process (control of maps and tool outputs as well as process) and *trust* (time for interaction and transparency of information). Respect includes *local knowledge* and *flexi*-*scale* components. Equity includes *perspectives and concern for future generations* (ability to represent ambiguous futures), *conflict management* (recognition of legitimacy of counter claims) and *capacity building* (ease of training in techniques and local use by local actors). Competence includes *local manageability and local use* (ease of use by community), and Accountability includes *transparency* (actors’ involvement in all stages, simplicity and ease of understanding) and *accessibility* (take-up of the geo-information products).

## Materials and methods

### Visualization-supported dialogues

Stakeholders in the visualization-supported dialogues represented local government and administration as well as sectoral authorities, branch organizations, civil society organizations, agricultural and forest enterprises, and other organizations with interest in the future management of each of the four catchments. During the first stakeholder dialogue, a number of development pathways were identified that included one or more measures that support ecosystem services, e.g. improve wastewater treatment, remove structures that hinder fish migration and fertilizer reduction. The interactive visualization tool was designed and developed to support stakeholder dialogues. These dialogues were designed to ensure that the tool could be used to display images and information to initiate discussions, facilitate and inform specific issues, contrast viewpoints or consequences of suggested governance actions and to identify and explore cost-effective and spatially explicit measures. The tool was used during stakeholder dialogues in the case areas (2016–2017), at a cross-case workshop (September 2017), and for data exploration during the Baltic Sea Region Workshop (November 2017). This paper draws on the analysis of material from the dialogues at the cross-case workshop. At this workshop, participants from the four case areas had access to the tool to explore pathways and scenario results of interest when discussing the overarching question: What opportunities do the various pathways offer and what barriers exist that hinder multi-functional ecosystem services? The dialogues were designed so that stakeholders from Selke and Berze were grouped in one discussion session and those from Reda and Helge in another session. Each session, with 2–3 stakeholders from each case area, lasted approximately 2 h, with focus on each of the areas in succession followed by a cross-case comparison and discussion of different effects and challenges. Facilitators initiated the sessions by introducing significant findings from each area, whereafter stakeholders could use the tool to visualize information that they wanted to explore and contrast to inform their discussions. The sessions were recorded and then transcribed. Notes were taken at the workshops to record when stakeholders or facilitators made use of the tool, e.g. illustrated the pathways and measures, viewed information for the entire catchment or specific sub-catchments, referred to, or queried the information or how it was calculated.

### The BONUS MIRACLE tool

**Fig. 1:**
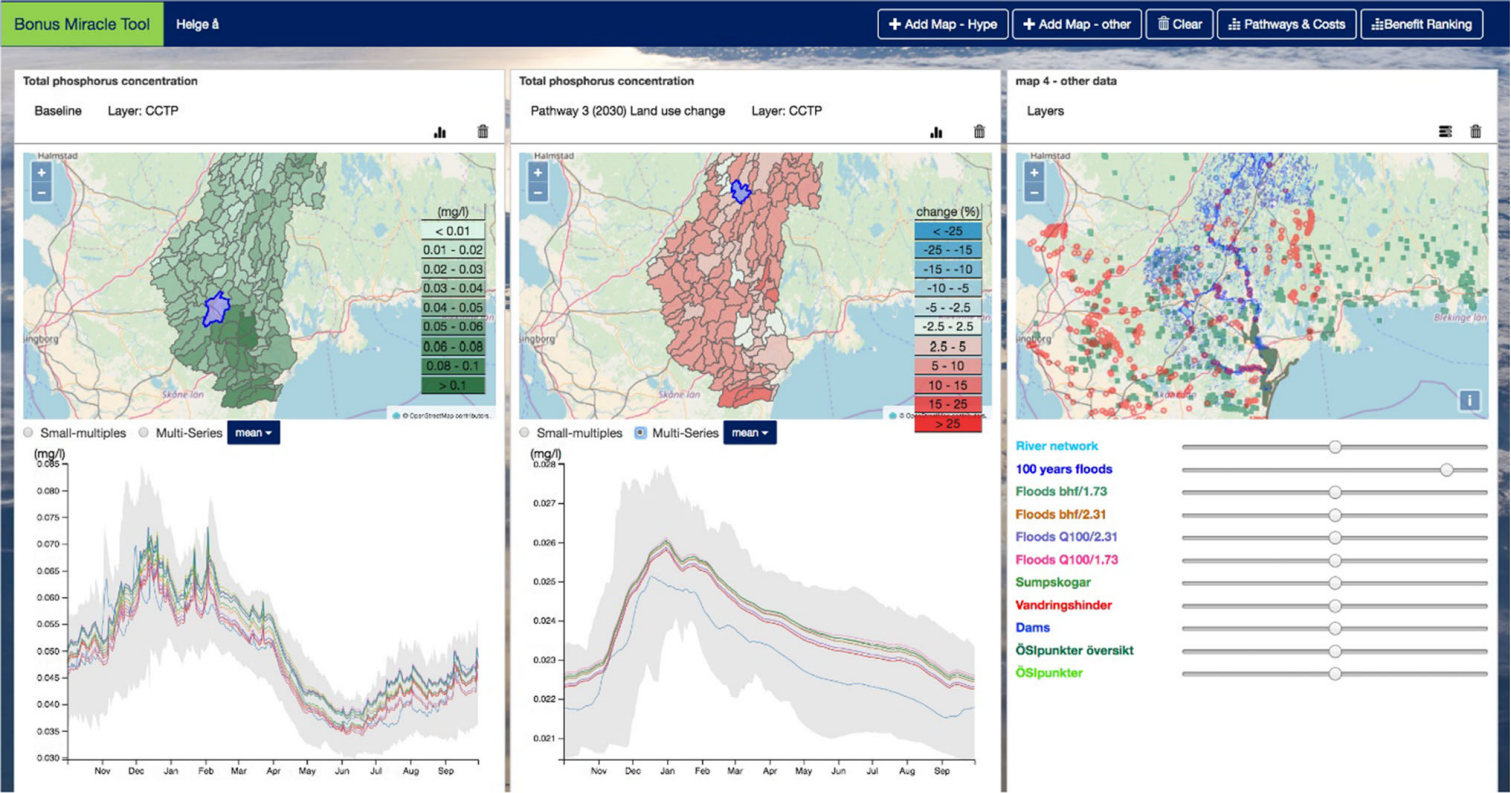
Example of the case area module for Helge catchment in Sweden, featuring two maps with the Total P concentration indicator (comparing Baseline data with Pathway 3 (2030) including land use change), comparing two different sub-basins in the multi-series graph below. The third window (map 4—other data) features supporting data with the interactive option to increase/decrease opacity for each of the layers

Tool development was guided by a number of parameters such as the profile of the stakeholders that were involved in the dialogues, the type of data that was relevant to the dialogues, and the chosen scenarios and pathways that were identified in the project. The tool (Fig. 1) provides information that stakeholders could make use of to inform their discussions and compare effects and costs of pathways and measures at different spatial and temporal scales. The tool allows users to select data sets, and view the modelled hydrological and hydro-chemical effects of different development pathways e.g. business as usual, ecosystem services approach, improved forestry management or improved waste water treatment, and climate and land use scenarios on nutrient concentrations and discharge as well as the estimated costs of the pathways and included measures and an interactive assessment of their benefits (ranked from low to high). The tool design includes a number of complementary views to support alternative ways of data exploration. The tool is composed of (1) a map view with choropleth, region and point of interest overlays, (2) time series views including multiline and small multiple time series and (3) Sankey diagrams for representing pathway costs.

The use of hydrological models led to information being displayed in maps divided into catchments and sub-catchments. The original set-up of sub-catchments, the choice of data used in the hydrological model, as well as considerations regarding what can or cannot be modelled, influenced the results that could be displayed. The tool consists of two modules—one with a focus on the case areas and the other on the Baltic Sea Region. In the *case area module,* the maps section (Fig. 1), presents maps of hydrological and hydro-chemical data that show nitrogen and phosphorus concentrations in rivers and discharge at catchment and sub-catchment outlets for conditions under baseline, defined pathways (different for each case) for current climate and defined pathways (different for each case) under future climate. All defined pathways were calculated with climate change forcing data for the 2020 impact period (2011 to 2030) for the case studies and the 2050 impact period (2036–2065) for the BSR. Forcing data from two models from the EURO-CORDEX ensemble, the WRF-IPSL-CM5A-MR and RCA4-CanESM2, were used to cover the ensemble spread. Both were driven with the RCP8.5 scenario (Representation Concentration Pathway 8.5 which implies + 8.5 Watts/m^2^ radiative forcing in the year 2100 relative to pre-industrial values). Baseline results representing the current conditions including all approved measures were calculated using reference periods of 2001–2020 for the case studies and 1991–2010 for the BSR. All climate change forcing data were bias-corrected with WATCH Forcing Data—ERA INTERIM (WFDEI) reanalysis forcing data. The values shown in the modelling tool represent long-term averages (20- and 30-year periods for the case studies and BSR, respectively) from two different climate models. The longer, 30-year periods were used for the BSR due to longer nutrient travel times and accumulations throughout the larger catchments. A range of values representing 25th through 75th percentiles from the time series, combining the results for both climate models, is shown in the graph charts for selected sub-catchments.

**Fig. 2:**
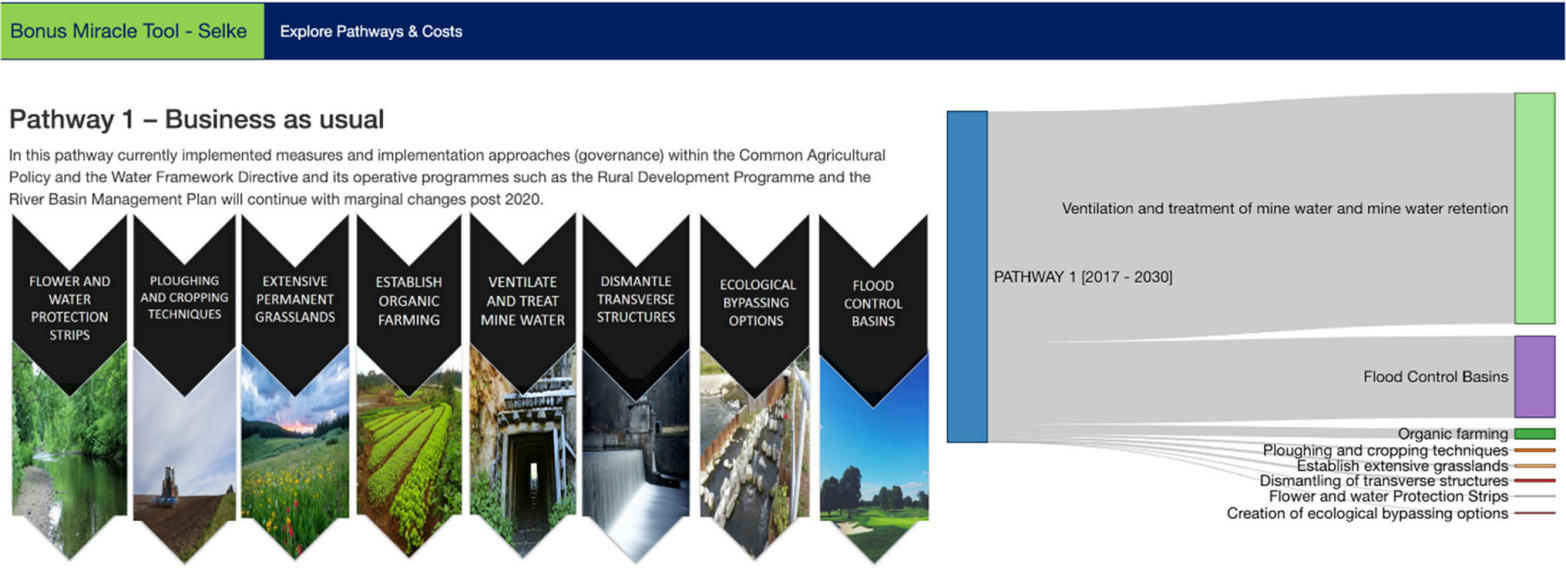
Example of the ‘Pathway and Costs’ for the Selke catchment in Germany. The figure shows the images representing the measures included in the pathway ‘Business as usual’, and the Sankey diagram showing costs

In the section ‘Pathways and Costs’ (Fig. 2) images that represent the measures included in each of the defined pathways are presented. Adjacent to each of the pathway images, a Sankey diagram illustrates the cost of the pathway and the separate costs of each measure.

**Fig. 3:**
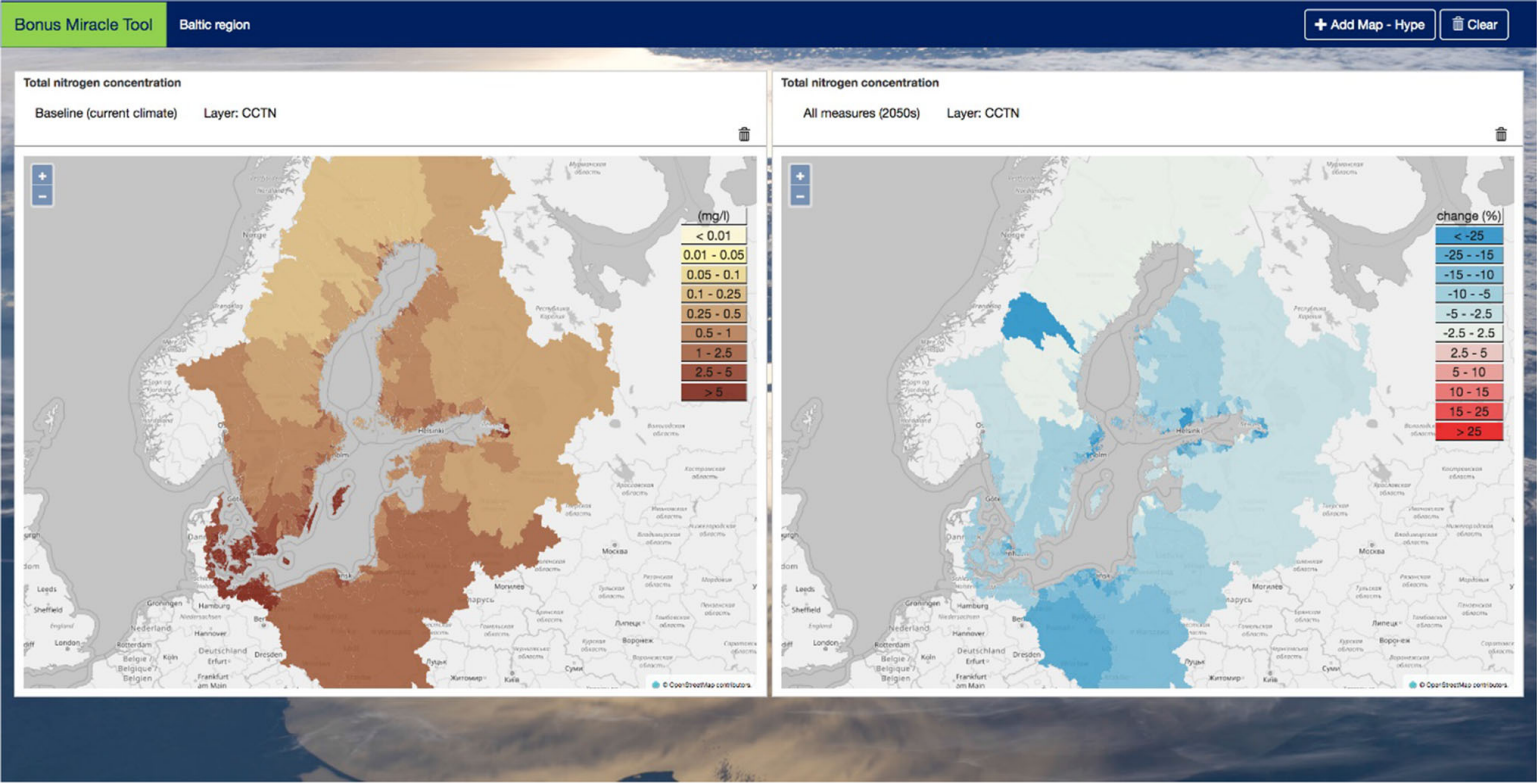
The Baltic Sea Region Module of the MIRACLE visualization tool showing ‘Total nitrogen concentration’ for the baseline (current climate) to the left and the relative change under future climate in the 2050s to the right. The two maps shown side-by-side allow simultaneous exploration of scenarios and indicators

The *Baltic Sea Region module* (Fig. 3) integrates input data and results generated with the E-HYPE model (Donnelly et al. 2016; Hundecha et al. 2016) that was set up and run for the 7156 catchments within the Baltic Sea drainage basin (Bartosova A. et al. 2017). It displays the effects of different measures on nutrient concentrations and flows from the 512 basins that directly drain into the Baltic Sea. The full results at catchment resolution, as well as details of the input data and model set-up, can be found in Bartosova et al. (2017, 2019). Results can be displayed for current conditions (baseline), a number of future scenarios representing climate change and selected Shared Socio-economic Pathways (SSPs 1, 2 and 5), or for scenarios that present results as a relative change from the baseline. The SSPs were defined after Zandersen et al. (2019) and represented within the model context. The indicators for the current baseline (current conditions including climate) and for the SSP2 baseline (SSP2 conditions with 2050s climate) are displayed directly as simulated by E-HYPE, e.g. the actual concentration of total nitrogen simulated at the outlet of the main stream.

## Results

We organized examples of the tool’s design and content, dialogue design and how the tool was used in the cross-case workshop dialogues according to the selected evaluation criteria—legitimacy, respect, equity, competence and accountability (McCall and Dunn 2012), that could contribute to good governance in the context of this study’s visualization-supported dialogues. We summarize these examples in Table 1 and discuss them in more detail in the sections below.

**Table 1:** Principles and components of Good Governance, adapted from McCall and Dunn (2012), and examples that demonstrate how they were addressed and/or attained in the stakeholder dialogues. “Joint” refers to the shared participation of stakeholders and researchers. “Stakeholders” refers solely to workshop participants outside of the research team

Principles	Components	Examples from the MIRACLE visualization-supported dialogues that support or hinder the principles
Legitimacy	Participation	Stakeholder involvement in defining pathways and influencing the focus of the dialogues
	Empowerment	Design of Baltic Sea Region knowledge platform Design of workshops to support cross-case learning
	Ownership	Joint process ownership Lack of stakeholder ownership of the data/information in the tool
	Trust	Inclusion of experts/modellers at workshops to increase understanding and clarity
Respect	Flexi-scale	High flexibility for multiple displays and transition between different spatial and temporal scales
	Local knowledge	Use of local knowledge to define pathways and assess and validate results
Equity	Future generations	Representation of jointly defined pathways Results for future scenarios of climate change and land use, as well as selected Shared Socio-Economic Pathways (SSPs)
	Conflict management	Platform design for exploring alternative pathways Dialogue format to encourage opposing perspectives
	Capacity building	Presentation of the tool and some outcomes to stimulate individual online use
Competence	Local use and manageability	Online format for laptop, smartphone or tablet use
Accountability	Transparency	Selection of mapped results Inspirational imagery to describe pathways
	Accessibility	Open access tool Inability to directly access/download the tool data/results English was the tool and workshop language

### Legitimacy

#### Participation

*Participation* in this study meant involving expert stakeholders in a number of organized dialogues so they could identify key issues related to e.g. water quality, water management, or ecosystem services of concern in each catchment and discuss challenges and ways to address them. These issues informed the chosen pathways and measures and guided the focus of the tool design, in terms of content and functionality, and dialogue design by influencing the set-up of the dialogues in terms of interaction with the tool as well as the information that was presented and discussed at the workshop. At the cross-case workshops, stakeholders used the tool to actively choose which results were viewed for different sub-basins to inform discussions. While Berze catchment stakeholders discussed the relationship between slope and phosphorus reduction on agricultural land, Selke catchment stakeholders commented on the usefulness of the tool for presenting accumulated effects of measures at the catchment outlet, in contrast to specific sub-basins under different land use.

#### Empowerment

Learning is an important component of *empowerment*. By providing modelled results of the effects of different pathways on river flow and nutrient loads, as well as the costs of specific measures, stakeholders had access to information that could increase the collective level of knowledge about eventual trade-offs affecting different management choices. During the dialogues, Reda catchment stakeholders pointed out the low effects of specific measures on downstream nutrient loads and questioned to what degree eventual costs are justified. The dialogue design with the two case stakeholder groups in each session allowed cross-case discussions and learning about differences in local preconditions related to, for example, nutrient concentrations and flows, policy and regulations, economic support, or potential willingness of farmers to undertake potential measures.

#### Ownership

Participants’ *ownership* of the process was exemplified in their identification of key issues in their catchments which guided the tool’s content and dialogue focus. The pathways and measures for each case were not pre-determined at the outset of the project, but specified by stakeholders at the first workshops. During the cross-case dialogues, images that illustrated the pathways and measures (see Fig. 2) were shown to introduce each case in order to review the outcomes of previous dialogues and create an easily understandable common base that would be explored in more detail during the dialogues.

#### Trust

Stakeholders in this study were professionals representing organizations with environmental interests and as such all were to some extent familiar with viewing and interpreting modelled results on maps. They were also somewhat aware that models do hold limitations and uncertainties related to the availability and accuracy of input data and the ways in which measures are translated into the model set-up, all which ultimately affect what can be modelled and the accuracy of the results. This meant that stakeholders could trust the visualized information, while also accepting its inherent uncertainty and limitations. At the cross-case dialogues, modellers were present and played a key role in helping to interpret results when stakeholders asked for more clarity and guidance. For instance, when Helge catchment stakeholders questioned results on the relationship between re-establishment of wet forests and peak flows, modellers explained how this measure was simulated in the model. They also pointed out that a lack of monitored records against which they could calibrate their model runs affects the uncertainty of the results.

### Respect

#### Local knowledge and flexi-scale

Local knowledge was of key importance in the definition of relevant pathways and measures for each area. The dialogue allowed stakeholders to relate their knowledge of the areas to the modelled results. Mapped results showed the relationship between fertilizer reductions and the lowering of nutrient concentrations under agriculturally classified land in Berze catchment. Stakeholders further clarified in which areas fertilized crops are grown, and agreed that these areas would show positive effects from such a measure. Such clarifications indicate how local knowledge can change the interpretation of modelled data and how the tool and dialogues enhance one another.

The interactive map-based tool allowed easy transitions between spatial scales and enabled up to three maps to be viewed beside one another to more easily assess different combinations of features and pathway results. Multiple, linked windows facilitated comparisons of different temporal scales at a chosen spatial scale. Helge catchment stakeholders made use of this feature in the cross-case dialogues to display and discuss effects of restoring wet forests on river flow under current and future climate.

### Equity

#### Future generations

The identification of future pathways by the stakeholders and the inclusion of modelled effects of various measures until 2030 under different climate change and land use scenarios highlight the relationship between current decisions and future effects. The inclusion of the effects on nutrient concentrations and flows under different climate change and land use scenarios and a number of Shared Socio-Economic Pathways allowed exploration of and reflection on multiple future scenarios and combinations representing ambiguous futures.

#### Conflict management

When exploring the change in nutrient loads after introduction of riparian zones or under reforested areas, Selke and Reda stakeholders discussed landowners’ divergent priorities and claims depending on economic considerations or lack of trust in modelled results at aggregated scales, to what degree it would be possible to implement the suggested measures, and what additional information such stakeholders might require. They also discussed how the Sankey diagrams display costs at catchment scale arguing that landowners might require other levels of aggregation to give meaning to these numbers.

### Competence

#### Local manageability and local use

The tool and the stakeholder dialogues were designed with specific consideration of different professional competences of the participants and their interests and priorities. The tool was available online prior to the dialogues, so stakeholders had the opportunity to explore and use the tool to inform and prepare discussions.

At the cross-case dialogues, the high complexity of the tool, including a significant number of pathways and scenarios containing up to seven indicators, presented a challenge for stakeholders to access the information themselves in the time frame of the sessions.

### Accountability

#### Transparency

The images that illustrated the suggested measures for each pathway aided *transparency* of the dialogues’ focus, as images represent a large amount of information that users can grasp more quickly than from reading text (Tufte 1997; Gershon and Ward 2001). The modelled results presented in map format allowed stakeholders to geographically orientate and choose results of specific pathways, measures and scenarios. More detailed information about the data, model structure, and which measures could be included in the Hype model were not presented in the tool. As such, some aspects of the tool’s content were not highly transparent, and the presence of researchers and modellers at the dialogues was important to provide additional clarifying information about the modelling process to support transparency in the process.

#### Accessibility

The tool is web-based so that it was openly accessible to stakeholders via the project website, before and during the dialogues. The operating language of the tool was English which might have limited its accessibility for some users. The modelled information as well as cost estimates was available via the tool during the dialogues, although data could not be directly downloaded or accessed. The foreign language combined with technical and academic wording of texts in the tool might also have made the tool content less accessible in terms of comprehension. In particular, the use of specific terminology to describe pathway measures could have led to misunderstandings, but could also have sparked dialogues to clarify the terms and hence support the development of a joint understanding.

## Discussion

### Barriers to achieving good governance in visualization-supported dialogues

At the cross-case dialogues, participants were professionals and represented organizations with environmental interests, and were hence somewhat familiar with results from hydrological models shown for sub-catchments and knowledgeable about the limitations and opportunities that models contain. However, questions did arise about the results and how the pathways and measures were represented in the model. It was important to have the modellers present for transparency and to increase trust and ownership of the results. The tool and dialogues were specifically designed for expert stakeholders. To promote good governance among a wider set of stakeholder groups, including those that would implement measures, e.g. landowners, would require re-design of the tool and the dialogue process to meet their interests, perspectives and expectations and to ensure information is provided at relevant spatial scale and is clearly displayed and communicated to promote transparency and accessibility. Similarly, if the tool and dialogue design were to be applied to a long-term deliberative process, continuous iteration would be required to ensure the inclusion of additional relevant data and novel perspectives, and thus build greater trust and accountability in the participatory approach (Turner et al. 2015).

For stakeholders to reach shared understandings of issues and approaches in environmental management, targeted efforts, insights and time are required (Salter et al. 2009; Mathevet et al. 2011; Sheppard 2012; Rød et al. 2015; Neset et al. 2019), as tools with high levels of complexity constrain direct user interaction if time is limited. User-specific instructions and sufficient allotted time to explore and reflect upon different combinations of information at different scales and time periods are needed to make sense of the tool and its content, depending on the level of expertise and familiarity of the user. While the amount of information and the degree of interactivity enabling users to move between different geographical and temporal scales, as well as view results of pathways, scenarios and measures of interest, were designed to increase ownership, transparency and engagement, it also led to a significant level of complexity that might have excluded some participants from parts of the dialogues. Besides tool complexity, having English as the tool and dialogue language in the cross-case dialogues might have further impacted on aspects such as legitimacy and accountability. Translating the tool’s text into local languages in further developments for usage among a wider audience and avoiding or re-wording technical terms would help rectify the transparency issue. A trade-off between complexity and comprehensive inclusion of data and information is difficult to avoid, and a balance must be discussed and decided upon according to context.

### Opportunities to support good governance in visualization-supported dialogues

The tool facilitated a comparative view of the different case areas as well as a comprehensive view of the entire Baltic Sea Region. When model results link choices with effects, including those on future generations, and sufficient time is allowed for reflection in stakeholder dialogues, this provides opportunities for experiential learning (de Kraker et al. 2011), and to enhance empowerment, legitimacy and equity. The tool’s usage during the cross-case dialogues demonstrated it to be a successful platform for presenting results to stakeholders and initiating discussions, and also for informing and contributing information into stakeholder-driven discussions to clarify and confirm specific findings. Model-based platforms have proved effective for enabling stakeholders to communicate, negotiate and integrate their views (de Kraker et al. 2011) and are an important step in knowledge production to enable people to make comparisons and synthesize their learning (Angelstam et al. 2013).

The flexibility of the tool allowing the display of results at multi-temporal and spatial scales gave stakeholders opportunities to share experiences, debate conflicting perspectives and negotiate perspectives and results as well as juxtaposing these with local knowledge to create a more comprehensive joint understanding of multiple perspectives and alternative pathways for different sites in the BSR. In this sense, collaborative learning could not only take place among expert stakeholders but also between and with the researchers, a characteristic linked to strengthening knowledge production and addressing long-term sustainability goals (Angelstam et al. 2013). As the tool in its current form did not contain more detailed information about the HYPE model used to generate results, the researchers and modellers present in the stakeholder dialogues were important knowledge brokers for providing clarity and increasing transparency. The cross-case dialogue design enabled stakeholders with different disciplinary and professional backgrounds and responsibilities to explore specific information related to their interests and case areas and convey and contrast this with that of others to enrich discussions and support transparency and ownership of the process.

## Conclusions

This study, based on a visualization-supported dialogue design, identified a number of barriers and opportunities to support good governance among expert stakeholders. While the specific examples were derived from participatory processes with expert stakeholders in four case areas around the Baltic Sea, the conclusions of this study can inform the wider field of participatory visualization research where visualization tools are designed and used to support and inform stakeholder dialogues. We conclude that in dialogues that include tools with a high degree of complexity, the presence of experts is essential for providing additional information, explanations or alternative approaches, to help overcome barriers in understanding and foster more informed interpretation of results. While several key components of good governance, such as participation, empowerment, accessibility and flexibility, were supported by the form and functionality of the tool, dialogue design was more instrumental in contributing to trust, inclusion of local knowledge and competence. The integration of a tool linking current choices with future effects and a dialogue design that allows time for stakeholder–tool interaction, reflection and discussions of results has the potential to contribute to empowerment, legitimacy and equity.
